# Determining items for inclusion in a decision support intervention for clinical trial participation: a modified Delphi approach

**DOI:** 10.1186/1745-6215-14-S1-O64

**Published:** 2013-11-29

**Authors:** Katie Gillies, Zoe Skea, Sara MacLennan, Craig Ramsay, Marion Campbell

**Affiliations:** 1 Health Services Research Unit, University of Aberdeen, Aberdeen, UK; 2 Academic Urology Unit, University of Aberdeen, Aberdeen, UK

## 

The use of decision aids in the context of decisions about trial participation is an emergent field. There is a lack of evidence about what information different stakeholders deem important for decisions about informed consent for clinical trials, and whether different groups agree on information. Therefore, the objective of this study was to determine items that different stakeholder groups view to be important for inclusion in a decision support tool for clinical trial participation; with a view to use these as a framework for developing decision support tools in this context.

A modified Delphi method was used to determine agreement on importance of items. The ‘expert' panel was made up of 49 individuals from 5 groups: 11 trialists; 6 research nurses; 7 ethics committee chairs; 9 decision support experts and 16 patients (9 trial experienced and 7 trial non-experienced). Two rounds of rating were completed. Items with a median of 7-10 with ≥65% of any one group in agreement were considered important for inclusion.

The expert panel achieved consensus on the majority of items included (60/66), agreeing that these were important for inclusion in a decision support tool for trial participation. These included items covering: information about trial participation and standard care; information on the likelihood of receiving different treatments; information to help patients' determine what matters most to them; ensuring the information is balanced; guidance on how to make a decision; disclosure of any conflicts of interest; using plain language in the tool and guidance on the development process Some areas of divergence amongst the panel were also identified relating to the use of patient stories.

Agreement was obtained on a number of items, which could be used as a framework to develop tools to support decision making about participation in clinical trials.

